# Differential immunogenicity of BNT162b2 or ChAdOx1 vaccines after extended-interval homologous dual vaccination in older people

**DOI:** 10.1186/s12979-021-00246-9

**Published:** 2021-08-20

**Authors:** Helen Parry, Rachel Bruton, Christine Stephens, Kevin Brown, Gayatri Amirthalingam, Ashley Otter, Bassam Hallis, Jianmin Zuo, Paul Moss

**Affiliations:** 1 Institute of Immunology and Immunotherapy, University of Birmingham, Birmingham, B15 UK; 2 National Infection Service, Public Health England, Colindale, London, NW9 5EQ UK; 3 National Infection Service, Public Health England, Porton Down, Salisbury, SP4 OJG UK; 4 University Hospitals, Birmingham, UK

**Keywords:** Vaccination, SARS-CoV-2, COVID, Antibody, Cellular, mRNA, ChAdOx1, Meta-analysis

## Abstract

**Background:**

Several SARS-CoV-2 vaccines have shown clinical efficacy against Covid-19 infection but there remains uncertainty about the immune responses elicited by different regimens. This is a particularly important question for older people who are at increased clinical risk following infection and in whom immune senescence may limit vaccine responses. The BNT162b2 mRNA and ChAdOx1 adenovirus vaccines were the first two vaccines deployed in the UK programme using an 8–12 week ‘extended interval’.

**Objectives:**

We undertook analysis of the spike-specific antibody and cellular immune response in 131 participants aged 80+ years after the second dose of ‘extended interval’ dual vaccination with either BNT162b2 mRNA (*n* = 54) or ChAdOx1 (*n* = 77) adenovirus vaccine. Blood samples were taken 2–3 weeks after second vaccine and were paired with samples taken at 5-weeks after first vaccine which have been reported previously. Antibody responses were measured using the Elecsys® electrochemiluminescence immunoassay assay and cellular responses were assessed by IFN-γ ELISpot.

**Results:**

Antibody responses against spike protein became detectable in all donors following dual vaccination with either vaccine. 4 donors had evidence of previous natural infection which is known to boost vaccine responses. Within the 53 infection-naïve donors the median antibody titre was 4030 U/ml (IQR 1892–8530) following BNT162b2 dual vaccination and 1405 (IQR 469.5–2543) in the 74 patients after the ChAdOx1 vaccine (p = < 0.0001). Spike-specific T cell responses were observed in 30% and 49% of mRNA and ChAdOx1 recipients respectively and median responses were 1.4-times higher in ChAdOx1 vaccinees at 14 vs 20 spots/million respectively (*p* = 0.022).

**Conclusion:**

Dual vaccination with BNT162b2 or ChAdOx1 induces strong humoral immunity in older people following an extended interval protocol. Antibody responses are 2.9-times higher following the mRNA regimen whilst cellular responses are 1.4-times higher with the adenovirus-based vaccine. Differential patterns of immunogenicity are therefore elicited from the two vaccine platforms. It will be of interest to assess the relative stability of immune responses after these homologous vaccine regimens in order to assess the potential need for vaccine boosting. Furthermore, these findings indicate that heterologous vaccine platforms may offer the opportunity to further optimize vaccine responses.

**Supplementary Information:**

The online version contains supplementary material available at 10.1186/s12979-021-00246-9.

## Introduction

Vaccines against SARS-CoV-2 have proven highly efficacious against the development of symptomatic Covid-19 [1–3]. A range of different approaches for delivery of the spike protein immunogen have been developed including mRNA, adenoviral and protein platforms, and the relative immunogenicity of vaccines is an important consideration for development of optimal vaccine protocols.

The immune correlates of protection against COVID-19 are uncertain at the current time although the magnitude of the spike-specific antibody response appears to be an important determinant of vaccine efficacy [4–6]. The total spike-specific immune response correlates with the level of neutralising antibody activity in most cases and this latter determinant may itself be a critical factor for vaccine efficacy. In contrast, the relative importance of the spike-specific cellular immune response is currently uncertain. T cell responses are important in controlling the severity of respiratory infections and it is notable that SARS-CoV-2 vaccines are themselves much more effective against prevention of severe clinical outcomes compared to milder asymptomatic infection [7]. As such, T cell responses against spike protein may well play an important role in the control of severe disease.

A striking feature of the COVID-19 pandemic has been the relative vulnerability of older people [8] and as such it is essential that Covid-19 vaccines demonstrate high efficacy within this population. This represents a potential challenge as vaccine-induced immune responses are often suboptimal in older people [9]. In particular, humoral responses following influenza vaccination are reduced in older people and have necessitated the introduction of range of novel vaccine platforms [10]. The basis for this effect is uncertain but is likely to reflect the impact of immune senescence in which the functional efficacy of the immune system deteriorates with age.

A further variable in the delivery of Covid-19 vaccines is the time interval between the 1st and 2nd doses of the vaccine. The BNT162b2 vaccine is licenced for delivery with a 3 week interval between doses and this approach has been adopted in many countries around the world. In contrast, improved immune responses following an extended interval between ChAdOx1 doses have been demonstrated and typically an interval of between 8 and 12 weeks is now recommended. Interestingly, the kinetics of antibody development following the ChAdOx1 vaccine are also delayed compared to the mRNA platform. The UK and several other countries have elected to employ an ‘extended interval’ vaccine regimen in which the second dose of either the BNT162b2 or ChAdOx1 vaccine is delayed for up to 12 weeks following the first dose. This approach was instigated in order to increase population coverage of single vaccination and both modelling approaches and real world clinical data have indicated the success of this regime in reducing mortality rates across the population [11].

Interestingly, the magnitude of the peak spike-specific antibody response is increased by threefold after extended interval delivery of the BNT162b2 vaccine and suggests that consideration should be given towards extending the current 3-week interval between vaccines [12]. However, an extended interval approach was also associated with a reduction in the magnitude of the peak cellular immune response following second vaccine. At the current time there has not been, to our knowledge, a direct comparison of the immunogenicity of the BNT162b2 or ChAdOx1 vaccines when used in an extended-interval vaccine regimen.

In this study we compare spike-specific antibody and cellular responses in older people following dual vaccination with either BNT162b2 or ChAdOx1. We demonstrate that the mRNA vaccine elicits stronger serological responses whilst the adenovirus vector drives more robust cellular immunity. These findings have a range of implications for long term vaccine immune monitoring and design of optimal heterologous vaccine platforms.

## Methods

### Study design and participants

131 participants aged 80 years and older, and who were living independently, were recruited to study. Co-morbidities were permitted. Participants were recruited between the 29th December 2020 and 4th March 2021. Local primary care networks identified vaccinees aged 80 years and older who had received either the BNT162b2 or ChAdOx1 vaccines and who were not living in a residential or care home, or requiring assisted living. Participants were sent invitation letters to take part and contacted the research team directly if they wanted to participate*.* Following initial contact with the study team, participants were then given the participant information sheet and consented verbally over the phone. This was substantiated with written consent obtained at the first phlebotomy time point. Ethical approval was obtained from North West Preston Research Ethics Committee with favourable outcome (REC 20\NW\0240) and work was performed under the CIA UPH and conducted according to the Declaration of Helsinki and good clinical practice.

### Procedures

All donors received two doses of either BNT162b2 from BioNTech/Pfizer or ChAdOx1 nCoV-19 from Astra Zeneca. 54 donors received BNT162b2 (median age 83 years, range 80–96) and 77 had received ChAdOx1 (median age 83 years, range 80–98). Younger healthy controls were also recruited following identification from primary care records and a letter invitation (29 donors received the BNT162b2 (median age 69 years, range 42–79) and 55 received the ChAdOx1 (median age 72, range 51–79) (Demographics in Table 1). A phlebotomy sample was taken at 2–3 weeks afer the second vaccine and acted as the primary time point for analysis. A matched sample taken at 5 to 6 weeks after the first vaccine was also available for comparative assessment. Samples were used for antibody and cellular studies.

**Table 1:** Patient demographics

	BNT162b2 elderly cohort	ChAdOx1 elderly cohort	BNT162b2 younger cohort	ChAdOx1 younger cohort
**Number of participants**	54	77	29	55
**Number with Nucleocapsid-specific Antibody detected**	1	3	3	5
**Median age**	83	83	69	72
**Age Range**	80–96	80–98	42–79	51–79
**Age IQR**	81–84	81–86	57–75	70–73
**Male**	24	33	12	18
**Female**	30	44	17	37
**Time since second vaccine (Median days)**	20 (IQR 18–21)	19 (IQR 18–20)	25 (20–33)	28 (19–32)

### Roche Elecsys® electrochemiluminescence immunoassay (ECLIA)

Total antibodies specific to SARS-CoV-2 were detected using electrochemiluminescence assays on the automated Roche cobas e801 analysers based at Public Health England (PHE) Porton. Calibration and quality control were performed as recommended by the manufacturer. Anti-nucleocapsid protein (NP) antibodies were detected using the qualitative Roche Elecsys® AntiSARS-CoV-2 ECLIA (COV2, Product code: 09203079190), whilst anti-spike (S) antibodies were detected using the quantitative Roche Elecsys® Anti-SARS-CoV-2 S ECLIA (COV2 S, Product code 09289275190), as previously described [13]. Anti-nucleocapsid results are expressed as cut-off index (COI) value, with a COI value of ≥1.0 considered positive for anti-nucleocapsid antibodies. Anti-spike results are expressed as units per ml (u/ml), with samples with a result of ≥0.8 U/ml considered positive for anti-spike antibodies within the fully quantitative range of the assay: 0.4–2500 u/ml. Samples > 2500 u/ml were diluted further (1:10, 1:100 and 1:1000) to within the quantitative range.

### Cellular assays

Peripheral blood mononuclear cells (PBMCs) were isolated from a whole blood sample using ‘T-Cell Xtend’ (Oxford Immunotec) and Ficoll. After quantification and dilution of recovered cells, 250,000 PBMC were plated into each well of a ‘T-SPOT Discovery SARS-CoV-2’ kit (Oxford Immunotec). This is designed to measure responses to overlapping peptides pools covering protein sequences of four different SARS-CoV-2 antigens, without HLA restriction, and includes negative control and PHA-stimulated cells as a positive control. Peptide sequences that showed high homology to endemic coronaviruses were removed from the sequences, but sequences that may have homology to SARS-CoV-1 were retained. Cells were incubated and interferon-γ secreting T cells were counted. A cut off of 24+ spots per million on the S1 pool was defined as a positive result in line with the Oxford Immunotec diagnostic Covid kit.

### Statistical analysis

Data were tested for normality using Kolmogorov-Smirnov analysis. For comparative analysis of antibody titres and cellular responses between BNT162b2 or ChAdOx1vaccine cohort, Mann-Whitney U-test or Wilcoxon for paired samples was performed. Antibody titres and T cell responses are presented as the median and IQR. Antibody titres are also presented as the geometric mean with SEM in supplementary Table 1. All analysis was performed using Graphpad prism v9.1.0 for Mac (San Diego, California USA).

## Results

### Spike-specific antibody responses are 2.9-fold higher after dual vaccination with BNT162b2 compared to ChAdOx1 in older donors

**Fig. 1:**
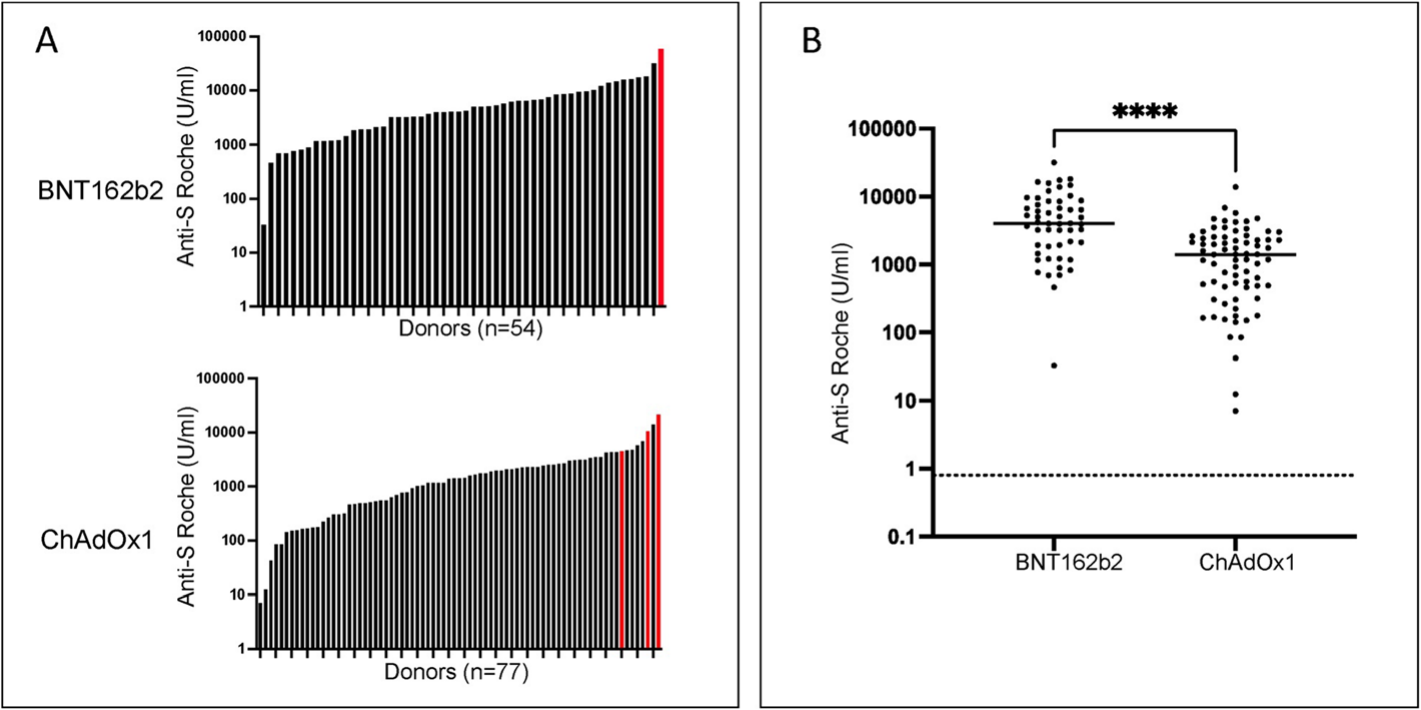
Antibody responses following second vaccination with either BNT162b2 or ChAdOx1. Data are shown as bar chart of total cohort (**A**) and dot plot excluding those with natural infection (**B**). Donors with previous natural infection are shown in red on the bar chart

131 donors, aged 80+ years age and who were living in the community, were recruited to the study. Blood samples were taken at 2–3 weeks after the second vaccine. Spike-specific antibody responses were assessed using quantitative Roche spike-specific ELISA and positive responses were seen in all donors after the BNT162b2 (*n* = 54) or ChAdOx1 (*n* = 77) vaccine respectively (Fig. 1). However, the median spike-specific antibody level was 2.9-times higher in donors who had received BNT162b2 vaccination. Specifically, median values were 4100 u/ml after BNT162b2 (IQR 1912–8613) compared to 1416 (IQR 480–2643) in ChAdOx1 vaccinees. Exclusion of 4 donors with serological evidence of previous SARS-CoV-2 infection, which is known to strongly boost vaccine responses [14], led to values of 4030 u/ml and 1405 u/ml respectively.

**Fig. 2:**
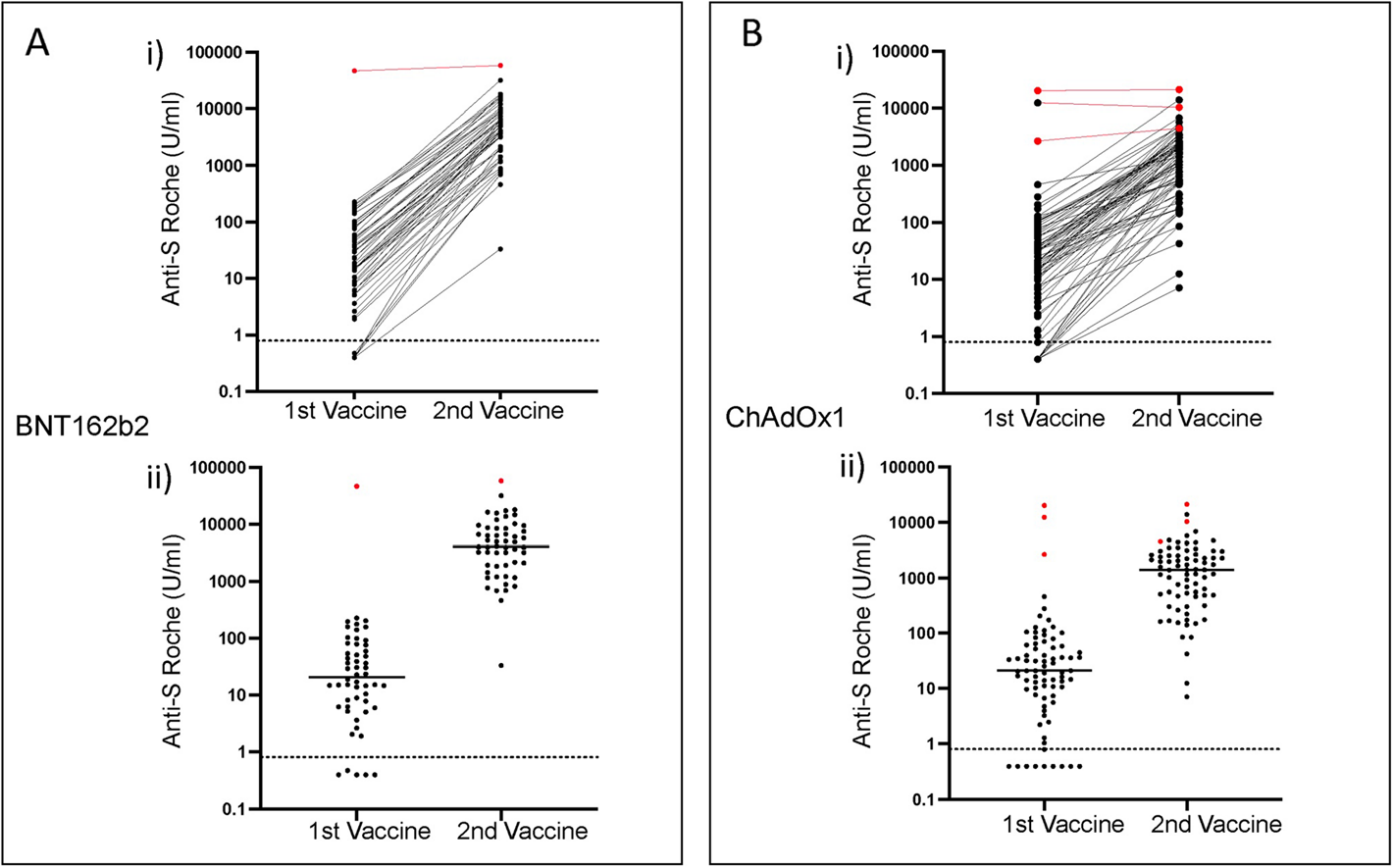
Antibody responses following 1st and 2nd vaccination with either BNT162b2 (**A**) or ChAdOx1 (**B**). Donors with previous natural infection are shown in red. (i) Paired individuals responses. (ii). Dot plot of whole cohort

**Fig. 3:**
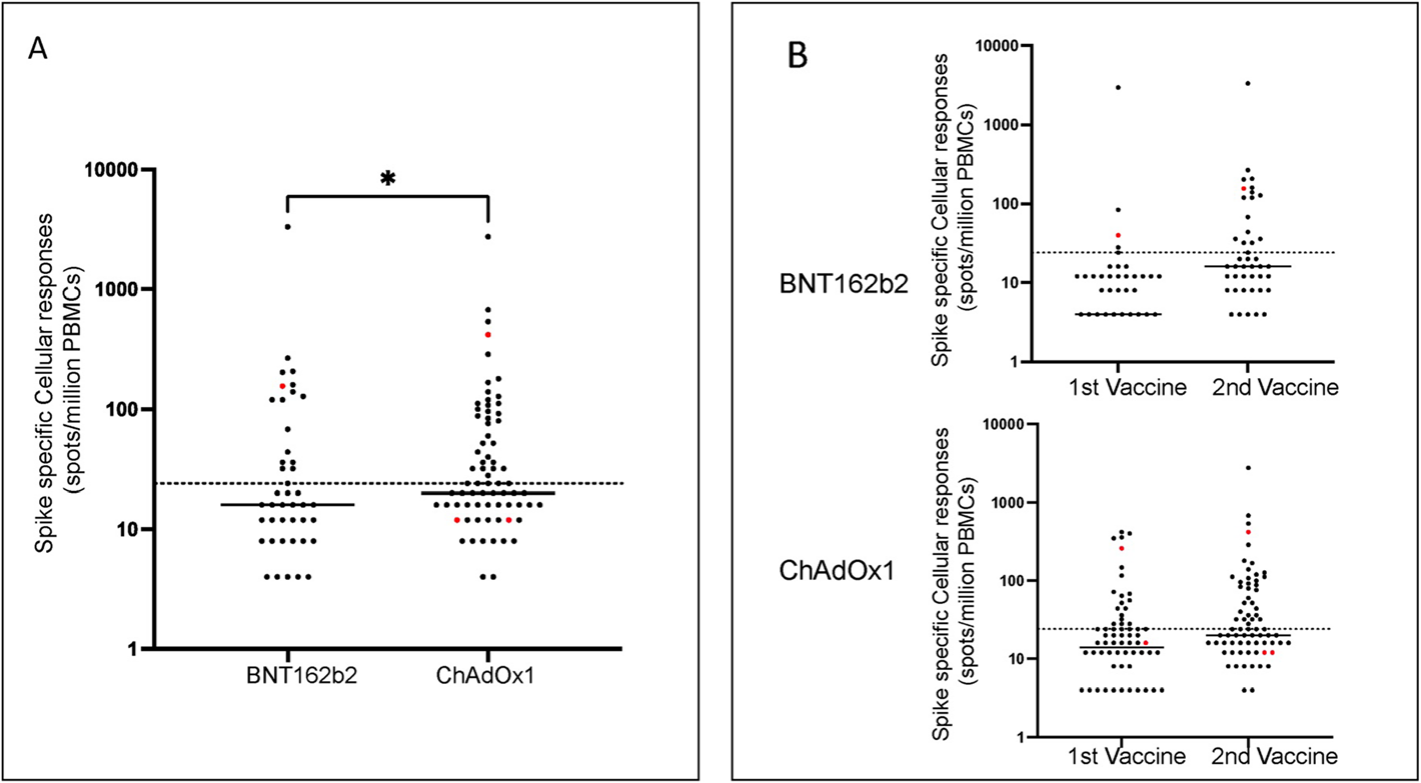
Spike-specific T cell responses after vaccination in donors aged 80+ years. (**A**) Spike-specific cellular responses following second vaccine with either BNT162b2 or ChAdOxl vaccine measured by ELISpot. Donors with previous natural infection are shown in red. (**B**) Comparison of cellular responses after first or second vaccine in relation to vaccine subtype

**Fig. 4:**
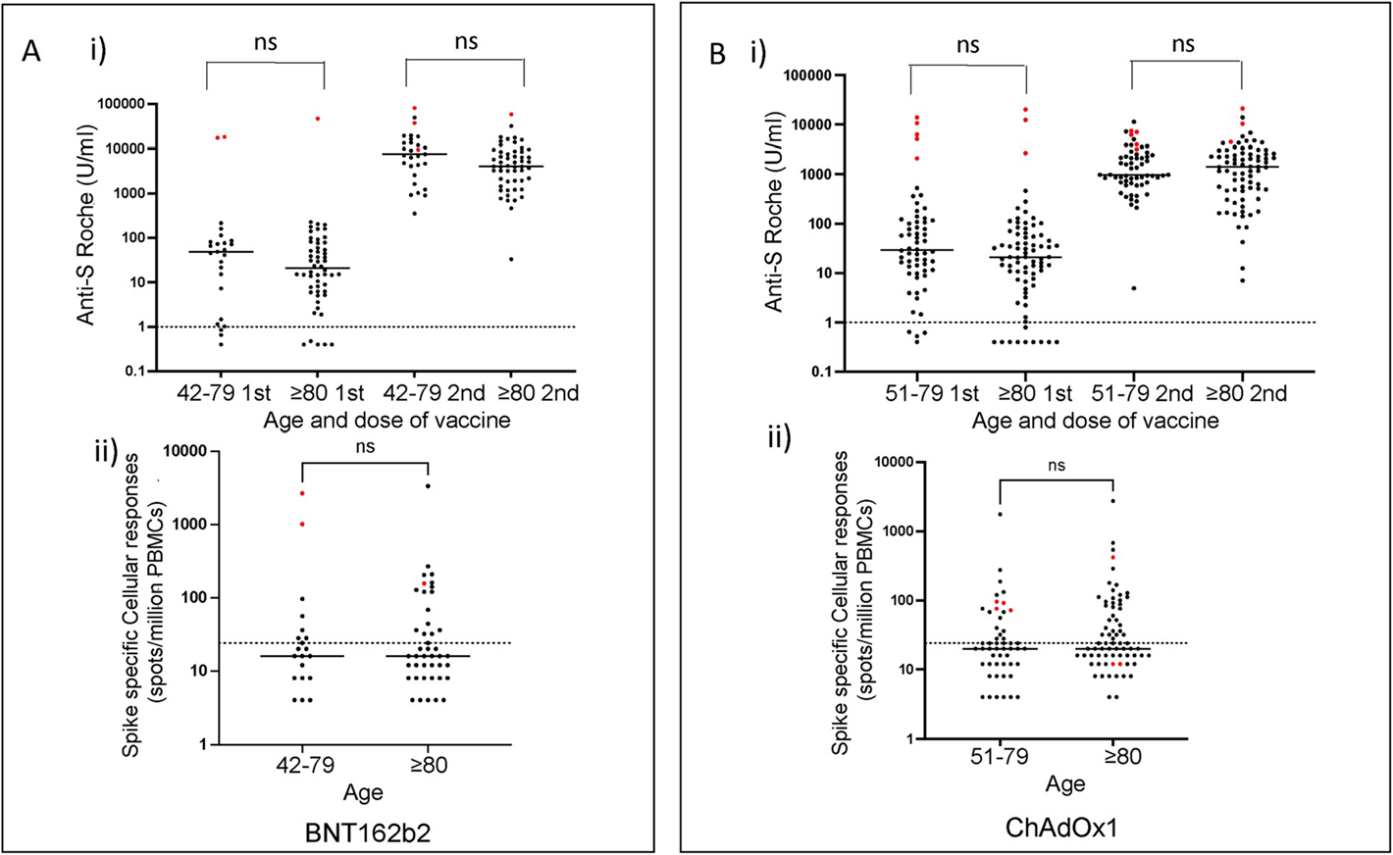
Spike-specific vaccine responses in younger and older cohorts. Responses following single or dual vaccination with **A**) BNT162b2 or **B**) ChAdOx1 in donors aged 80+ or 42–79 years. i) Spike-specific antibody responses by age and by vaccine dose. ii) Spike-specific Elispot response following second vaccine

### Elevated spike-specific antibody responses after dual BNT162b2 vaccination reflect enhanced incremental response to the second vaccine

In addition to blood samples taken after the second vaccine we also had access to matched patient samples that had been taken at 5–6 weeks after the first vaccine. Antibody and cellular analysis of these samples has been reported previously and demonstrated that antibody levels were comparable after a single dose of either BNT162b2 or ChAdOx1 vaccine [13]. As such we next went on to determine the pattern of incremental antibody response after the second vaccine within this cohort.

For all BNT162b2 vaccinees the median antibody level was 21 after the first vaccine (IQR 6.2–76.5) which rose to 4100 after the second vaccine. As such the booster vaccine led to a 197 fold increase in antibody response (Fig. 2A). One donor had serological evidence of previous SARS-CoV-2 infection and here these values were 47,100 and 58,800 respectively. Exclusion of this donor allowed assessment of vaccine responses in 53 infection-naïve donors where median antibody responses were 19 (IQR 6.2–68) and 4030 (IQR 1890–8530) respectively after the first and second vaccines, representing a 214-fold increase (*P* < 0.0001).

For ChAdOx1 vaccinees, paired samples were available for 77 donors of whom 3 had serological evidence of previous infection with SARS-CoV-2. Within the total cohort median antibody levels rose from 21 (IQR 7–60) to 1416 (IQR 480–2640) between first and second vaccines, a 67-fold increase *p* < 0.0001 (Wilcoxon) (Fig. 2B). For the three donors with previous infection these values were 12,400 (IQR 2680–20,200) and 10,350 (4500–21,240) respectively. Values within the 74 donors without previous infection were 20 (IQR 6.4–53) and 1405 (IQR 470–2540) respectively, representing a 70-fold increase (*p* < 0.0001).

These data show that the second dose of either vaccine strongly boosts antibody responses. However, within infection-naïve donors this relative increment is three-fold higher with the BNT162b2 vaccine which explains the higher final responses after dual vaccination.

### Spike-specific cellular responses are 1.4-fold higher after dual vaccination with ChAdOx1 compared with BNT162b2

Our interim analysis of immune response at 5 weeks after first vaccine in this cohort had shown higher spike-specific cellular responses after the ChAdOx1 vaccine [14] and we next assessed relative responses after dual vaccination using interferon-γ ELISpot.

Within BNT162b2 vaccinees, 51 of the 54 donors had paired cellular responses for analysis. The proportion of donors who showed a cellular response above the threshold rose from 9.8% (5/51) to 33.3% (17/51) after first and second vaccination respectively (Fig. 3A). The median level of response increased to 14 spots per million PBMC after excluding the single donor with previous natural infection (*P* < 0.0001) (Fig. 3B).

For donors who received ChAdOx1 vaccination, paired T cell results were available for 76 of the 77 donors. The proportion of positive responses increased from 34.2% (26/76) after the first vaccine to 48.6% (37/76) after second vaccine (Fig. 3A). After excluding donors with evidence of natural infection, the median absolute count was 20 spots per million after first and second vaccine (*p* = 0.0014) (Fig. 3B).

These findings indicate that ChAdOx1 stimulates a stronger spike-specific cellular response in older people although the relative incremental advantage of this vaccine above BNT162b2 falls from 3-fold to 1.4-fold between the first and second doses of vaccine.

### Spike-specific immune responses after dual vaccination in donors aged 80+ years are comparable to those seen in younger donors

Vaccine-induced immune responses are often suboptimal in older people and this may reflect the impact of immune senescence. As such, we then compared our findings in donors aged 80 years and above with a population of donors aged between 42 and 79 years of age in whom antibody and cellular responses were measured in a similar way.

Of note, immune responses at both timepoints were comparable between both cohorts with no significant impairment of antibody or cellular responses in older people. In donors without previous natural infection, median antibody responses after BNT162b2 for younger and older cohorts were 46 and 19 after the first vaccine (*p* = 0.57) and 7026 and 4030 after second vaccine respectively (*p* = 0.1) (Fig. 4A). For ChAdOx1 the comparable values were 25 and 20 (*p* = 0.08) and 949 and 1405 respectively (*p* = 0.96) (Fig. 4B). These results indicate a trend towards slightly lower antibody responses after dual vaccination with BNT162b2 in older people.

T cell assays were available after the second vaccine in the younger cohort. Within infection-naïve donors the median T cell responses after BNT162b2 in the older and younger cohorts was 14 spots/million in both groups whilst after ChAdOx1 these values were equivalent at 20 spots/million.

As such these findings show that dual homologous vaccination with either BNT162b2 or ChAdOx1 is highly effective for induction of spike-specific immune responses in older people and largely overcomes any significant effect of immune senescence.

## Discussion

Determination of the immune correlates of Covid-19 vaccination is critical for guiding deployment of optimal vaccine regimens within the population. This analysis is particularly important in older people for whom the mortality risk from Covid-19 infection is markedly increased. In this report we show that dual vaccination with the BNT162b2 or ChAdOx1 vaccines elicits distinct profiles of immune response in older people. These observations raise a number of questions regarding the underlying basis for these differences and how they may relate to clinical application.

A striking feature of the work was that spike-specific antibody responses were observed in every donor following dual vaccination with either vaccine. This is highly encouraging in relation to the immunogenicity of these vaccines in older people and is likely to underlie the strong clinical protection against severe Covid-19 infection that has been observed. The magnitude of the spike-specific antibody response is emerging as an immune correlate of vaccine efficacy and if responses are maintained over the longer term then these findings augur well for protection in this group.

It was noteworthy that the peak antibody responses were nearly 3-times higher in recipients of the BNT162b2 vaccine compared to those who had received the ChAdOx1 vaccine. Our previous report within this cohort demonstrated equivalent antibody responses at 5-weeks after the first ‘priming’ dose of each vaccine. As such, this differential effect after the second dose dose reveals that an incremental antibody boost to the second dose is the key factor that distinguishes the mRNA and Adenovirus-based platforms. Indeed, the median increment was 214 for BNT162b2 compared to 70 for the ChAdOx1 vaccine. These findings may reflect the strong humoral immunogenicity of mRNA delivery which induces germinal centre responses and development of spike-specific memory B cells. It will be important to assess how these antibody responses develop and are maintained over time as studies have shown that immune responses are somewhat delayed after the ChAdOx1 vaccine compared to BNT162b2. As such it is possible that the disparity in titres will change over time. There has been some concern that immune responses against the adenoviral vector might act to limit the degree of boosting from this vaccine but it was clear that a strong incremental response was still seen after the second ChAdOx1 dose.

One feature of the analysis was that the relative increment in antibody responses after the second vaccine was very similar between donors. This was particularly striking within the BNT162b2 regimen and shows that assessment of antibody response after the first vaccine is a strong predictor of ultimate response. The reasons that underlie the differential response to the vaccine within the population are uncertain but it will be of interest to see if these features are maintained after a third booster dose.

In contrast to humoral immunity, spike-specific cellular responses were somewhat less marked after dual vaccination. A previous report has shown that cellular responses after single vaccination are stronger with the ChAdOx1 vaccine and this advantage was maintained after two vaccines. Boosting of the T cell response was 1.7-fold and 3.5-fold higher after the ChAdOx1 and BNT162b2 vaccines respectively and as such the relative differential in cellular response between vaccines was reduced from 3-fold after the first vaccine to 1.4 fold following the second. It will be of interest to assess if any difference is maintained after booster vaccination. T cell responses were measured with an interferon-γ Elispot assay and although this is a standard technology in this setting it will be important in future work to assess the relative response from CD4+ and CD8+ T cells subsets and differential cytokine profiles. The underlying basis for enhanced cellular response after ChAdOx1 is not clear but is likely to reflect the nature of antigen presentation from the adenoviral vector expression system [15].

The potential significance of the differential cellular immune response is not yet clear but could impact on both the longevity of humoral immunity and also clinical efficacy [16]. It will therefore be of interest to assess the temporal stability of antibody response within the two cohorts and correlate this with cellular immune profile. Furthermore, although cellular responses are likely to help to limit the severity of Covid-19 infection, the rates of clinical protection with mRNA-based vaccines are at least as high as those seen with ChAdOx1.

Given the differential profile of immune responses following the two vaccines it is tempting to speculate that a combinatorial heterologous vaccine regime might offer superior immunogenicity compared to homologous delivery [17]. Of note, superior cellular responses have been seen with a ChAdOx1/BNT162b2 regimen compared to standard-interval homologous BNT162b2 vaccination [18] and this heterologous regime mediated enhanced cellular priming whilst maintaining robust humoral immunity with a 4-week vaccine interval [19]. It will be of interest to await results of heterologous ChAdOx1/BNT162b2 combination using a ‘delayed-interval’ vaccine regimen.

Immune responses following vaccines such as influenza are often suboptimal in older people [20, 21], and as such were interested to assess spike-specific immunity within different age groups. Interestingly, we did not find any differences in the antibody or cellular response between this cohort of people over 80 years of age and a group of 96 donors between 42 and 79 years of age. These findings are in line with the excellent clinical protection against Covid-19 that is afforded following vaccination in older people and indicate that the nature of the spike immunogen, or the novel mRNA and ChAdOx1 delivery platforms, appear able to overcome any serious impact from immune senescence [22]. However, some negative influence of age has been seen in other studies [23] and it will also be important to assess the functional quality of these immune responses with assays such as neutralisation or cellular cytotoxicity. No influence of gender on vaccine-induced immune response was apparent although larger population studies have revealed lower antibody responses in men [24].

A limitation of our study was the fact that we do not have detailed information on co-morbidities of donors in the study.

In conclusion, ‘extended-interval’ dual vaccination with either BNT162b2 or ChAdOx1 elicits strong spike-spike specific immune responses in older people which is consistent with real world effectiveness data. The two vaccines exhibit differential ability to induce antibody or cellular responses and this may become important in assessment of future combination regimens.

## Supplementary Information

**Additional file 1: Supplementary Table 1.** Antibody responses presented as the geometric mean following first and second vaccine in participants aged 80 years and older without evidence of previous infection.
