# Supplementary material for: Differential immunogenicity of BNT162b2 or ChAdOx1 vaccines after extended-interval homologous dual vaccination in older people
Source: Immun Ageing. 2021 Aug 20;18:34. doi: 10.1186/s12979-021-00246-9 (PMC8377354; doi:10.1186/s12979-021-00246-9)
Supplement: Supplementary file 1 — Additional file 1: Supplementary Table 1. Antibody responses presented as the geometric mean following first and second vaccine in participants aged 80 years and older without evidence of previous infection. [file 12979_2021_246_MOESM1_ESM.docx]

**Supplementary table 1**

Antibody responses presented as the geometric mean following first and second vaccine in participants aged 80 years and older without evidence of previous infection.
